# DNA Methylation Biomarkers for Young Children with Idiopathic Autism Spectrum Disorder: A Systematic Review

**DOI:** 10.3390/ijms24119138

**Published:** 2023-05-23

**Authors:** Andrea Stoccoro, Eugenia Conti, Elena Scaffei, Sara Calderoni, Fabio Coppedè, Lucia Migliore, Roberta Battini

**Affiliations:** 1 Department of Translational Research and of New Surgical and Medical Technologies, University of Pisa, 56100 Pisa, Italy; 2 Department of Developmental Neuroscience, IRCCS Stella Maris Foundation, 56128 Pisa, Italy; 3 Department of Neuroscience, Psychology, Drug Research and Child Health NEUROFARBA, University of Florence, 50139 Florence, Italy; 4 Department of Clinical and Experimental Medicine, University of Pisa, 56126 Pisa, Italy

**Keywords:** autism spectrum disorder, epigenetics, DNA methylation, idiopathic ASD, peripheral biomarkers

## Abstract

Autism spectrum disorder (ASD) is a complex neurodevelopmental condition, the underlying pathological mechanisms of which are not yet completely understood. Although several genetic and genomic alterations have been linked to ASD, for the majority of ASD patients, the cause remains unknown, and the condition likely arises due to complex interactions between low-risk genes and environmental factors. There is increasing evidence that epigenetic mechanisms that are highly sensitive to environmental factors and influence gene function without altering the DNA sequence, particularly aberrant DNA methylation, are involved in ASD pathogenesis. This systematic review aimed to update the clinical application of DNA methylation investigations in children with idiopathic ASD, investigating its potential application in clinical settings. To this end, a literature search was performed on different scientific databases using a combination of terms related to the association between peripheral DNA methylation and young children with idiopathic ASD; this search led to the identification of 18 articles. In the selected studies, DNA methylation is investigated in peripheral blood or saliva samples, at both gene-specific and genome-wide levels. The results obtained suggest that peripheral DNA methylation could represent a promising methodology in ASD biomarker research, although further studies are needed to develop DNA-methylation-based clinical applications.

## 1. Introduction

Autism spectrum disorder (ASD) represents a complex neurodevelopmental condition, the basic symptoms of which include alterations in social functioning and in communication, as well as repetitive behaviors and restrictive interests [1]. The global prevalence of ASD is rapidly increasing and is currently estimated to affect 100 in 10,000 children, with a male–female ratio of 4.2 [2]. Even though ASD symptoms begin to appear early during toddlerhood, the diagnosis is still usually made at 3–5 years of age, thus preventing a prompt early intervention and related optimal outcomes [3,4]. The currently available tools for ASD diagnosis are mainly based on clinical behavioral evaluations, and biological markers that could potentially support early diagnosis are lacking [5].

The aetiopathogenesis of ASD is complex and has yet to be completely understood. Several genetic and environmental factors that are potentially implicated in ASD etiopathology have previously been identified [6]. The pivotal role played by genetic factors is well documented by the high heritability observed in twin studies, which has been estimated to be 64–91% [7]. Moreover, several genetic syndromes can manifest impairments associated with ASD, including Angelman, Fragile X, and Rett syndromes, as well as tuberous sclerosis [8]. It is now clear that the genetics underlying ASD is complex, as numerous genomic variations, including copy number variants (CNVs), de novo mutations, and single-nucleotide polymorphisms (SNPs), have been identified in ASD individuals [9]. With over 1000 genes reported in the SFARI (Simons Foundation Autism Research Initiative) Gene database, and over 3000 ASD-associated genes in the AutismKB database, the number of genetic variations found in people with ASD is continuously growing [10,11]. Candidate genes for ASD are implicated in different pathways that are fundamental for typical neurodevelopment, including neural synaptic communication, the regulation of gene expression, immunity, and inflammation [12,13].

Nevertheless, causative genetic variants seem to account for only 10–20% of ASD cases; meanwhile, in at least 70% of ASD individuals, the pathophysiology remains largely hidden. In these cases, the etiology of ASD is probably characterized by the interplay between low-risk genes and exposure to adverse environmental cues [14]. Indeed, a large number of studies suggest that prenatal and newborn exposure to various environmental factors make a significant contribution to the ASD risk. These factors include advanced parental age, maternal obesity, maternal immune system disorders, prenatal exposure to air pollution, premature birth, low birth weight, and caesarean delivery [15,16]. However, it is not yet clear which is the causal link between environmental exposure and the disturbance of neurodevelopment that underpins ASD.

Epigenetics is emerging as an important player in mediating the effects of environmental factors on genomic regulation in several human pathologies [17]. Epigenetic mechanisms, including DNA methylation, histone tail modifications, and non-coding RNA activity, are able to alter gene function without changing nucleotide sequences [18]. Several pieces of evidence suggest that epigenetic mechanisms, particularly DNA methylation, are involved in ASD pathogenesis [19]. It is worth noting that several risk genes for ASD play a role in chromatin regulation and are part of the epigenetic machinery [20]. For example, some syndromic forms of ASD are due to mutations in genes involved in DNA methylation establishment and reading, such as the *MECP2* gene in Rett syndrome [21]. Indeed, the *MECP2* gene encodes for the Methyl-CpG-Binding Protein 2, a protein that binds methylated DNA and, in turn, regulates the expression of several genes throughout the genome [22]. Regarding idiopathic ASD, altered DNA methylation has frequently been identified in different tissues of ASD patients, including post mortem brain tissue, placenta specimens, and peripheral blood [23]. Although investigations of the post mortem brain can provide relevant knowledge about the pathophysiology underlying ASD, the availability of brain tissue samples is limited, and these samples mainly reflect the epigenetic landscape of modifications in older subjects who present with well-established symptomatology. On the other hand, more accessible tissues, including saliva or peripheral blood, could be used for DNA methylation investigations; they could constitute useful biomarkers, improving our understanding of the pathophysiological changes associated with ASD progression [24]. Indeed, it is known that methylation levels in peripheral tissues can provide peripheral marks that can be used as a proxy for molecular alterations of the central nervous system in living patients [25]. Potentially altered DNA methylation in the peripheral blood of ASD children was already suggested in a research published in 2004, in which ASD children were shown to have altered plasma levels of the metabolites required for DNA methylation reactions, including methionine, S-Adenosyl methionine, and homocysteine, thus reflecting a metabolic profile consistent with an impaired capacity for DNA methylation [26]. The interest in finding a DNA methylation signature as a biomarker with clinical relevance to enhance ASD management is shown by the increase in the amount of research on this topic in recent years. However, it is not yet clear whether peripheral blood DNA methylation biomarkers are available for the diagnosis or assessment of the symptomatic severity of ASD.

**Figure 1:**
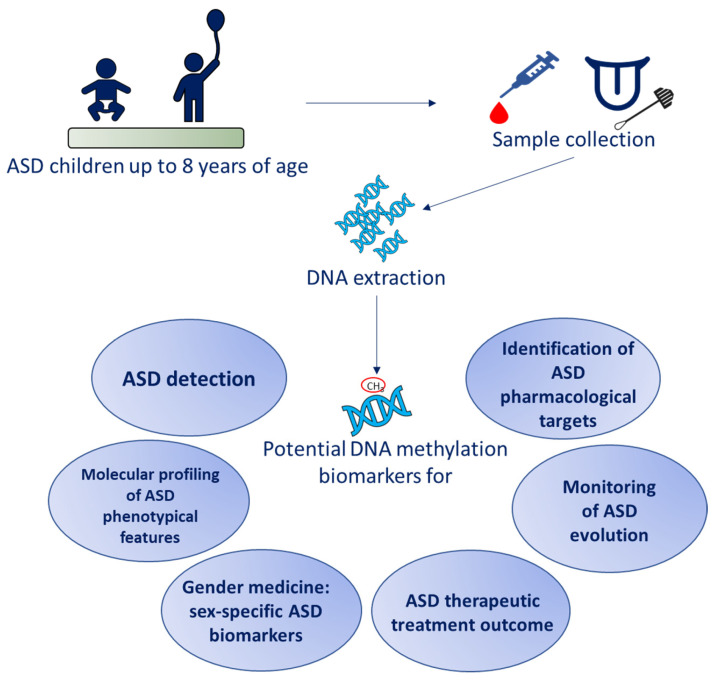
Rationale of the study.

The current systematic review aims to update the clinical application of DNA methylation investigations in children with idiopathic ASD, focusing on its implication in clinical diagnosis, prognosis, and the outcomes of therapeutic treatments (Figure 1). Since early diagnosis is highly important in this field, we narrowed the search to include young children (below eight years of age). We selected studies focused on children with a mean age of under eight years for the following two reasons: (i) to minimize the confounding effects of patient development and puberty status on DNA methylation [27,28]; and (ii) to reduce the impact that different variables (e.g., environmental factors, psychiatric comorbidities, and psychotropic medication) and their interactions may have had on the patients’ epigenetic profiles. Considering the significant impact that age has on epigenetic expression in every subject, we focused on the young population in order to limit related bias and to extrapolate information that may be helpful in anticipating diagnosis, even in milder cases. To this end, a literature search was performed on various scientific databases using a combination of terms related to the investigation of DNA methylation in the peripheral tissues of children with idiopathic ASD.

## 2. Materials and Methods

### 2.1. Literature Research

The literature search was performed using three databases, PubMed, Scopus, and Web of Science, following the Preferred Reporting Items for Systematic Reviews and Meta-Analysis (PRISMA) guidelines [29]. We focused our systematic review on the relevant literature that aimed to investigate DNA methylation patterns in the peripheral tissues of young children with ASD. According to the PICO strategy, we defined the following criteria to answer the review question: (P) participants—children with idiopathic ASD; (I) intervention—evaluation of DNA methylation in easily accessible peripheral tissues; (C) comparison—not applied, and (O) outcome—associations between DNA methylation analyses and ASD status and/or ASD clinical presentations. The terms used were: “DNA methylation” AND “autism spectrum disorder” OR “ASD” AND “children” OR “toddler”. Moreover, the reference lists of the included articles and of literature reviews focused on DNA methylation and autism were checked. The latest database search was conducted in May 2023. Reviews were used to gather original studies but were not included in the selection process. After the removal of duplicates, the articles resulting from the search had to meet the following criteria to be included in the current review.

### 2.2. Eligibility Criteria

The inclusion and exclusion criteria, established before the literature search, were as follows:

Inclusion criteria:

- Studies focused on DNA methylation
- Studies including individuals with a well-established diagnosis of idiopathic ASD
- DNA methylation analyses in children with a mean age ≤ 8 years
- Studies performed on easily available peripheral tissue (i.e., peripheral blood, saliva, buccal swabs)
- Availability of the full text of the paper

Exclusion criteria:

- Articles published in languages other than English
- Reviews and/or meta-analyses
- Studies performed on DNA from the placenta or cord blood
- Non-peer-reviewed studies
- In vitro and in silico studies or studies using animal models

No discrimination regarding the study design or the method used to investigate DNA methylation was applied. Two authors independently screened the literature (A.S. and E.C.). Any inconsistencies were resolved by group discussion. We excluded studies performed on placenta and cord blood samples given the difficulty of implementing this technique in routine clinical practice. Moreover, as a maternal–foetal organ, the placenta’s pattern methylation does not necessarily reflect that of the child’s DNA [30], and there is the possibility that DNA extracted from cord blood could be contaminated by maternal DNA [31].

### 2.3. Assessing the Risk of Bias, Outcome Assessment, Quality Scoring, and Statistical Methods

Two authors (A.S. and E.C.) performed the quality assessment independently, and any disagreement between them was resolved with the other authors. The risk of bias was evaluated using the “Study Quality Assessment Tools” available at the US National Institute of Health [32], selected in accordance with the design of the included studies: observational cohort and cross-sectional studies, case–control studies, and studies with no control group. Due to high heterogeneity in the study designs, the methodology for DNA methylation evaluations used, and the different means of reporting the results, it was not possible to perform a quantitative pooling of the existing data.

## 3. Results

### 3.1. Study Selection

**Figure 2:**
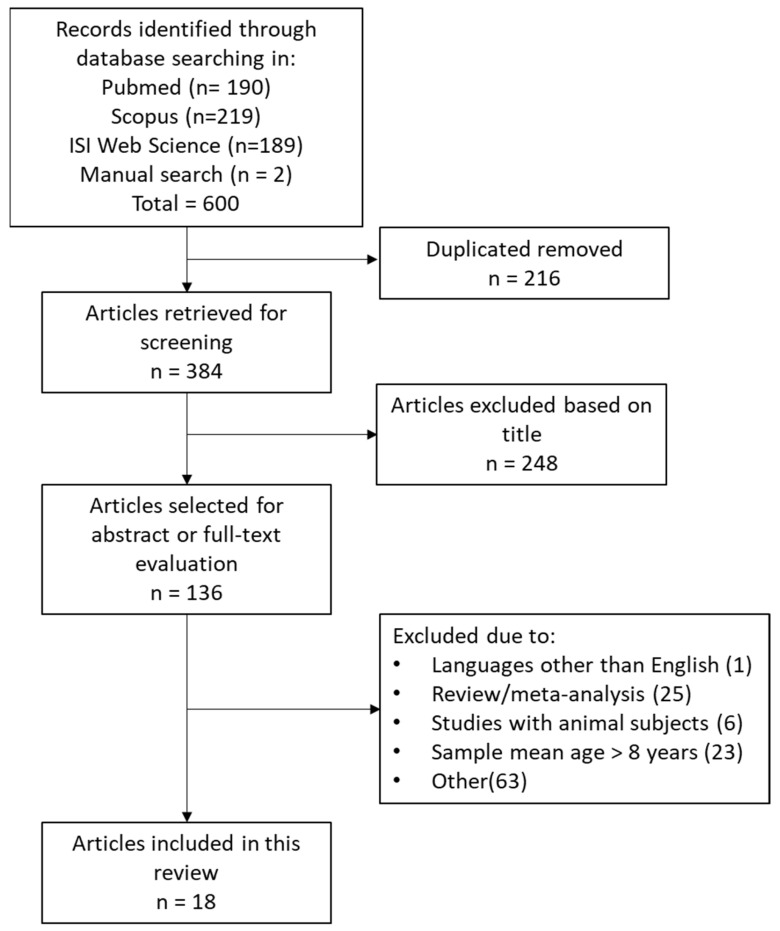
PRISMA flow chart of the literature search and study selection.

Details of the study selection process and exclusion criteria are shown in the PRISMA diagram (Figure 2).

Overall, 190 papers were identified from PubMed, 219 from Scopus, and 189 from Web of Science. Two additional studies were identified manually. After the removal of duplicates, 384 articles were further screened through title evaluation. On the basis of the titles, 136 articles were further screened for evaluation of the abstract or fulltext. A total of 18 studies met the inclusion criteria and were included in the review (Table 1).

### 3.2. Summary of the Included Studies

The main characteristics of the 18 studies included in the systematic review are reported in Table 1. Different study designs were applied across the 18 articles. Case–control studies were the most commonly used (*n* = 13), while a few papers examined participants longitudinally (*n* = 3), with samples collected before diagnosis or at multiple time points after diagnosis. In the majority of the studies, DNA was extracted from peripheral blood (*n* = 14); in 2 studies it was extracted from buccal swab cells, and in 2 studies it was taken from neonatal dried blood spots.

### 3.3. DNA Methylation Investigation Techniques

Global DNA methylation, epigenome-wide analysis, and candidate gene analysis are the three main approaches used to investigate DNA methylation [51]. Global DNA methylation provides an average of the total DNA methylation following the quantification of the methylcytosine content (5-mC) in the genome. Epigenome-wide association studies (EWAS) investigate methylation levels at specific CpG sites throughout the genome; they are typically hypothesis free and screen up to hundreds of thousands of loci across the genome. EWAS analyses reveal the differentially methylated positions (DMPs, i.e., CpG sites with statistically significant differences in the average methylation levels between groups) and differentially methylated regions (DMRs, i.e., differences in the mean DNA methylation in genomic regions including multiple consecutive CpGs sites) between cases and controls. On the other hand, the candidate gene approach aims to investigate DNA methylation levels in a limited number of genes based on an a priori hypothesis or to validate EWAS findings, i.e., genes that have been found to be associated with the disease in investigations at the genome-wide level. In 8 studies included in the current systematic review, DNA methylation was investigated at the whole-genome level (EWAS approach), and the method most frequently used was the Illumina 450 K array (*n* = 7), while the EPIC array and RRBS were used only in one study each. In one study, the EWAS approach was followed by a candidate gene approach, which confirmed the results. The candidate gene approach was used in eight studies. The techniques used for the candidate gene approach were pyrosequencing (*n* = 3), MS-HRM (*n* = 2), qMSP (*n* = 2), BSP (*n* = 2), and MSRE-PCR (*n* = 1). Global DNA methylation levels were investigated in three studies using an ELISA assay with antibodies against 5-mC, the HPLC technique, and LINE-1 methylation analysis.

### 3.4. Characteristics of Participants

According to our systematic search of scientific databases, all studies included in this review investigated DNA methylation marks in young children with Autism Spectrum Disorder (ASD), a clinical condition characterized by socio-communication impairments with repetitive interests and behaviors. Information regarding the mean age of participants, the sample size and gender ratio, and diagnostic/screening criteria for ASD were clearly reported in the majority of the papers, or were obtained from the supplementary information. Studies were not included in the review if age-related information was not available.

### 3.5. Main DNA Methylation Findings

In 8 of the 18 studies, the DNA methylation pattern was investigated at the genome-wide level. Notably, the 450 K array was the most commonly used technique. While this approach allows researchers to obtain a large amount of information on the methylation levels of a target genome, there is a need to identify strong differences between groups in order to determine genome-wide significance after applying a correction for multiple comparisons [52]. Therefore, some authors also reported results of “suggestive statistical significance”, i.e., significance levels established by the authors that do not reach the significance threshold for multiple comparisons in genome-wide analyses [34]. In line with this, an investigation performed on peripheral blood with the 450 K array failed to identify DMPs or DMRs at a genome-wide significance level but identified them using less stringent *p*-values [34]. Indeed, Andrews et al. did not identify DMPs or DMRs at a previously established Bonferroni correction level of *p* < 1.12 × 10^−7^, but they identified seven differentially methylated CpG sites at a statistically significant difference of between 1.74 × 10^−6^ and 7.20 × 10^−3^ between ASD and TD [34]. DMPs were associated with various genes, including *CENPM*, *FENDRR*, *SNRNP200*, *PGLYRP4*, *EZH1*, *DIO3*, and *CCDC181*. Interestingly, five of these sites, particularly those associated with the *CCDC181* gene, showed a direction of effect consistent with that previously observed in brain specimens from ASD patients [53]. Two studies used the 450 K array to search for DNA methylation levels in DNA extracted from buccal cells [35,40]. After correcting for multiple comparisons, Aspra et al. identified four significant DMRs between ASD and TD subjects; these were closely located to the *FAIM2* (hypomethylated), *CPXM2* (hypermethylated), *NRIP2* (hypermethylated), and *SOX7* (hypomethylated) genes. Moreover, two DMRs were identified from the interaction between ASD and sex. One of the DMRs encompassed the 5′UTR region of the *ZFP57* gene and was found to be hypermethylated in ASD individuals. The second DMR encompassed the 5′UTR region of the *GSTT1* gene and was hypomethylated in the ASD group compared to the TD one. Moreover, a target of *ZFP57*, the *RASGRF2* gene, was found to be hypomethylated in ASD individuals [35]. Gui et al. investigated whole-genome DNA methylation by means of the 450 K Array in buccal swab DNA from 51 male infants at 8 months, 10 of whom were diagnosed with ASD at 3 years of age. At a significance level of *p* < 5 × 10^−5^, 32 probes were significantly associated with ASD, of which two probes were in genes previously associated with ASD, including the *CACNA2D1* and *SND1* genes [40]. Two studies using the 450 K array were performed on DNA extracted from neonatal dried blood spots in children later diagnosed with ASD [36,41]. After adjusting for multiple comparisons, Bahado-Singh et al. identified 249 differentially methylated genes between 14 children who later developed ASD compared to 10 TD children. Some of the genes identified, including *EIF4E*, *FYN*, *SHANK1*, and *VIM*, were previously associated with ASD. The best CpG sites predictive for ASD development were associated with seven genes including *NAV2*, *OXCT1*, *LOC389033*, *MYL9*, *ALS2CR4*, *C19orf73*, and *ASCL2* [36]. Hannon et al. investigated the DNA methylome in the dried blood spots of 1263 neonates, of whom 629 were later diagnosed with ASD. At an EWAS threshold level, no DMPs were identified between the two groups. However, 20 ASD-associated DMPs were identified at a significance level of *p* < 5 × 10^−5^. The most significant CpG site associated with ASD was located in the 5′UTR region of the *RALY* gene. Moreover, 7 DMPs characterized by an interaction between autism status and sex were identified at a significance level of *p* < 5 × 10^−5^ [41]. A methylome analysis using the 450 K array and the RRBS was conducted with 4 pairs of concordant ASD twins, 5 discordant ASD individuals, and 30 pairs of sporadic patients with aged- and sex-matched controls [44]. In the first step, the authors performed an EWAS analysis of the 5 discordant ASD twins, identifying 2397 DMRs between the ASD and TD twins. Then, following a functional enrichment analysis, 35 genes were identified as promising candidates for influencing ASD pathophysiology. One of these, the *SH2B1* gene, was chosen for further bisulfite pyrosequencing analyses in all the subjects enrolled; its methylation levels were found to increase in ASD-discordant monozygotic twins compared to concordant monozygotic and in sporadic ASD compared to control subjects [44]. In one study, methylome data obtained in the peripheral blood of 67 ASD patients by means of the 450 K array were correlated with prenatal environmental risk factors, including maternal stress and gestational complications [46]. Several DMPs were identified in children of mothers with different grades of psychological stress vulnerability, although the names of the genes were not reported. Moreover, authors calculated the epigenetic clock as a proxy of postnatal stress exposure, finding that children had a higher biological age than chronological age [46].

Only one study used the EPIC array technique to investigate the methylome in DNA extracted from the peripheral blood of 23 ASD individuals, 23 Fragile X syndrome individuals with autism (FXSA), and 11 TD children [36]. By using a Benjamini–Hochberg multiple-test correction, 156, 79, and 3100 DMPs, and 14, 13, and 263 DMRs among TD vs. ASD, TD vs. FXSA and ASD vs. FXSA, respectively, were identified. Two genes that were differentially methylated between TD and ASD, namely, *PAK2* and *FANCD2*, were among those listed in the SFARI Gene database as being previously associated with ASD [43].

Three studies aimed to investigate global DNA methylation levels in children with ASD [33,39,45]. One of these studies observed that global DNA methylation levels evaluated by means of HPLC were lower in the peripheral blood of 68 ASD individuals than in the 54 TD and 40 unaffected siblings [38]. In a later study, global DNA methylation was assessed by evaluating the methylation of LINE-1 sequences using pyrosequencing [32]. The authors observed decreased methylation in the peripheral blood of ASD patients, particularly in cases with neurodevelopmental regression, when compared to TD individuals. In a recent paper, global DNA methylation levels, which were evaluated by means of an ELISA assay with antibodies against 5-mC, were shown to decrease in the neutrophils of 28 ASD children when compared to 24 TD children [33].

Gene-specific methylation analysis was performed in eight studies. Increased methylation of the *NCAM1* gene was observed in the peripheral blood of ASD children when compared to TD individuals, as well as increased *NGF* gene methylation levels in ASD with neurodevelopmental regression when compared to both TD and ASD without regression [39]. By using pyrosequencing, increased methylation of the *ST8SIA2* gene was observed in the peripheral blood of ASD compared to TD children [49]. In two reports, gene-specific DNA methylation was evaluated by means of MS-HRM [31,40]. Gallo et al. analyzed the methylation levels of the *MECP2*, *OXTR*, *HTR1A*, *RELN*, *BCL-2*, and *EN-2* genes in the peripheral blood of 42 ASD females; they observed that high maternal gestational weight gain was associated with increased *BDNF* methylation and that a lack of maternal folic acid supplementation and low *RELN* methylation was associated with more severe ASD [38]. The same genes were analyzed by Stoccoro et al. in the peripheral blood of 23 male and 35 female ASD individuals, and sex-related methylation differences were observed [47]. In particular, the methylation levels of the *MECP2*, *HTR1A*, and *OXTR* genes were connected to females, while those of the *EN2*, *BCL2*, and *RELN* genes were connected to males. Moreover, it was observed that various maternal factors, including a lack of folic acid supplementation, were associated with high disease severity; moreover, *BDNF* methylation levels were associated with advanced maternal age at conception, which, in turn, was connected to cesarean delivery and, through it, to various pregnancy complications and stressful events, all suspected pregnancy risk factors for ASD [47]. Only one study used the MSRE-PCR technique to perform gene-specific methylation analyses [37]. In that study, a higher frequency of *OXTR* promoter hypomethylation was observed in the peripheral blood of 27 ASD individuals compared to 39 TD individuals [37]. The qMSP technique was used in two studies [42,50]. Hu et al. investigated methylation levels of the *HTR4* gene in the peripheral blood of 61 ASD children and 66 TD children, and decreased methylation was observed in the ASD individuals. The difference was significant only for male subjects. Moreover, higher methylation was observed in female ASD individuals than in male ASD individuals, while the gender difference was not significant in TD subjects [42]. Zhao et al. investigated the methylation patterns of *TGFB1*, *BAX*, *IGFBP3*, *PRKCB*, *PSEN2*, and *CCL2* in the peripheral blood of 42 ASD patients and 26 TD patients, finding that the rate of the methylation of *TGFB1* was decreased in ASD and was positively associated with the interaction ability score [50]. One study used the BSP technique to investigate the methylation levels of the *ESR2* gene in the peripheral blood of 54 ASD and 54 TD individuals, observing that 8 CpG sites in the *ESR2* 5′-flanking region were hypermethylated in ASD subjects and that 4 CpG sites were positively associated with the severity of the symptoms [48].

Adjustments for age or sex were generally performed, and a few studies controlled for additional covariates such as bath effects and cellular heterogeneity. More information on the characteristics of the included studies is reported in Table 1.

Overall, the studies included in the current systematic review clearly show that DNA methylation is altered in the peripheral tissues of children with ASD; these findings could potentially provide biomarkers of clinical status, related severity, and exposure to adverse environmental factors. However, no gene was found to be altered in more than one study. The lack of replication could be due to several reasons, including different sample sizes, the different aims of the investigations, and the different techniques used to evaluate DNA methylation.

## 4. Discussion

The present review aimed to evaluate the potential use of DNA methylation as a peripheral biomarker to help clinicians in the assessment and treatment monitoring of ASD. Indeed, the diagnosis of ASD is currently mainly based on clinical and behavioral evaluations, and stable and non-invasive biomarkers that could help clinicians or even anticipate the diagnostic process are still lacking. In total, 14 of the 18 publications selected were based on peripheral blood investigations, 2 out of 18 were based on buccal cell samples, and 2 out of 18 analyzed neonatal dried blood spots. The papers included in the current systematic review suggest that DNA methylation in peripheral tissues could provide potential biomarkers for ASD. However, it should be noted that the evidence obtained before now does not yet allow for the identification of a valid epigenetic biomarker for ASD, as the most interesting findings need to be replicated by independent research groups to be confirmed. The lack of reproducibility of the findings could be due to several factors, including the different tissues used, the small study population sample sizes that are often used, the different genetic backgrounds of the individuals enrolled, the different study designs, and the different techniques used to investigate DNA methylation.

Several papers focused on DNA methylation alterations in ASD have been published, but many of them were not included in this review as they were conducted on the brain specimens of deceased patients, on placentas and cord blood, or on the peripheral tissues of individuals older than eight years of age. Indeed, we narrowed the search to young children to underline the importance of investigating neurobiological markers capable of identifying features of autism even in cases where the symptoms appear to be more evident by the beginning of primary school. This decision also controlled for development, puberty status, and other variables that can potentially have an impact on epigenetics [27,28].

A previous systematic review aimed to summarize the evidence of epigenetic involvement in neurodevelopmental disorders without using limitations in source tissue for DNA extraction or patients’ ages [54]; it indicated that the *OR2L13* gene, which encodes for an olfactory receptor, is a potential peripheral epigenetic marker for ASD, given that its methylation levels were found to be altered in two independent case–control investigations, one performed on buccal cells [55] and the other on peripheral blood [56]. It is notable that altered *ORL2L13* gene methylation was also identified in a paper included in the current review, which focused on a younger population [36]. Indeed, Bahado-Singh et al. identified increased *OR2L13* methylation in the dried blood spots of neonates who were subsequently diagnosed with ASD [36]. Interestingly, *OR2L13* has also been found to be differentially expressed in the brain samples of ASD patients [57], further suggesting its potential usefulness as an ASD peripheral biomarker and a target for understanding ASD etiology. Another gene that deserves to be mentioned is *BCL2*, the methylation levels of which were found by Liang et al. to be altered in peripheral blood [44]; they were also previously found to be altered in lymphoblastoid cell lines derived from ASD individuals [58]. Additionally, the methylation levels of the *ZNF57* and *SDHAP3* genes have been found to be altered in the ASD temporal cortex and cerebellum, respectively [53], and a paper in the current systematic review [36] identified the same genes as having altered methylation in the dried blood spots of neonates who later developed ASD.

In addition to helping in the identification of subjects with ASD, DNA methylation analyses could also provide useful information on the clinical expression of the condition. Gallo et al. observed that peripheral blood *RELN* gene methylation levels inversely correlated with ASD severity, as evaluated with the Autism Diagnostic Observation Schedule 2 (ADOS-2) assessment tool [38]. The *RELN* gene encodes for the reelin protein that is involved in neuronal migration during brain development and in synaptic plasticity in the adult brain [59]. Genetic mutations in the *RELN* gene and altered reelin pathways have been associated with several neurological disorders, including schizophrenia, bipolar disorder, depression, and ASD, and altered methylation levels of the *RELN* gene have been found in the postmortem brain tissues of post-puberal and adult ASD patients [59,60,61]. According to data obtained by Gallo et al. [38], the peripheral methylation levels of another gene, *ESR2*, have also been associated with ASD severity as evaluated by the Children’s Autism Rating Scale [48]. Another study included in the current review observed increased *NGF* (encoding the nerve growth factor) methylation levels in the peripheral blood of ASD individuals without mental regression during the first two years of life compared both to TD individuals and to ASD patients who develop mental regression [39]. A previous paper on the same ASD population identified altered nerve growth factor protein levels in the plasma of ASD individuals; more interestingly, they differed between ASD subjects with and without mental regression, suggesting that the methylation of *NGF* could modulate its expression [62]. In addition to disease severity, peripheral blood DNA methylation has been associated with the behavioral phenotypes of ASD. Indeed, the methylation of a CpG site of the *ST8SIA2* gene has been found to positively correlate with stereotyped behaviors [49], while the methylation of *TGFB1* was found to positively correlate with the interaction ability score [50]. Although the results of these studies are of great interest, as they suggest that peripheral DNA methylation could provide biomarkers to detect different levels of expression of the ASD spectrum, they need to be replicated in further studies.

Epigenetic investigations could also help us to understand the different prevalence of ASD in males and females. Indeed, ASD is diagnosed in about 4.2 males for every female [2]. Several theories have been proposed to explain the higher prevalence of ASD in males, including the “extreme male brain theory”, which hypothesizes that the autistic brain is a hypermasculinized male brain; the “camouflaging theory”, i.e., the ability of females to camouflage their symptoms and perceive them differently; and the “sex chromosome theory”, according to which the presence of two X chromosomes would be protective against ASD [63]. Whatever the mechanism underlying the sex ratio of autism, in recent years, it has been observed that the differences between males and females in terms of susceptibility to diseases, as well as in responses to adverse environmental factors, could rely on epigenetic mechanisms [64]. In line with this concept, a study included in the current systematic review showed that the methylation levels of some ASD-related genes are different between the two sexes in peripheral blood, with the *MECP2*, *HTR1A,* and *OXTR* genes more highly methylated in female ASD individuals, and the *EN2*, *BCL2*, and *RELN* genes more highly methylated in males [47]. Furthermore, another study found decreased *HTR4* methylation in the peripheral blood of ASD individuals, but the difference was significant only for male subjects [42]. Moreover, in buccal cells, two DMRs located in the proximity of the *ZFP57* and *GSTT1* genes were found between ASD and control subjects after considering males and females separately [35]. Similarly, seven DMPs have been identified in the dried blood spot DNA of neonates who were later diagnosed with ASD only after considering the interaction of DNA methylation with the sex of the children [41]. Additionally, a study performed on cultured lymphoblastoid cell lines derived from idiopathic ASD individuals and sex-matched siblings revealed that DNA methylation alterations between the two groups affect the genes of several important ASD-related pathways in different manners in females and males [65]. Overall, these three studies suggest that DNA methylation in peripheral tissues could differ between males and females, and sex should be carefully considered when searching for ASD-related DNA methylation biomarkers.

Three studies included in the current review that aimed to investigate global DNA methylation observed that the peripheral DNA of ASD subjects is markedly hypomethylated when compared to TD subjects [32,33,38]. It is worth noting that this finding resulted from three different research groups with three different techniques, thus strengthening the evidence that peripheral global DNA hypomethylation is a feature of ASD expression in young children. Moreover, global DNA methylation levels were found to be lower in severe ASD children when compared to mild–moderate ASD children, thus suggesting that the extent of DNA hypomethylation correlates with the extent of the disease severity [33]. Although these results are of interest, the clinical utility of the evaluation of global DNA methylation in the management of ASD children needs to be further investigated.

Of particular interest is the study by Alshamrani et al. [33], which reported that the decreased global DNA methylation in the neutrophils of ASD children was accompanied by increased inflammatory mediators, thus showing a direct link between dysfunctional epigenetics and immune and the inflammatory alterations that are frequently detected in ASD individuals. Indeed, both adult and young ASD subjects often show alterations in blood immune cells, including T cells, B cells, monocytes, granulocytes, and natural killers, as well as altered peripheral cytokines and chemokines, including interferon-gamma (IFN-γ) and interleukin-17 (IL-17) [66]. Another observation made in [33] is that decreased global DNA methylation in ASD could be induced by environmental pollutants that have epigenetic effects, including phthalates. Indeed, the authors observed that the neutrophils of ASD children treated with di(2-ethylhexyl) phthalate (DEHP) exhibited a marked reduction in DNA methyl-transferase activity, with a concomitant significant reduction of 5-mC levels when compared to the neutrophils of TD children treated in the same manner [33]. Moreover, the authors observed that DEHP treatment induced increased expression of two inflammatory mediators, namely, CCR2 and MCP-1, in the neutrophils of ASD, but not in TD children’s neutrophils; this effect can be prevented by the pre-treatment of the neutrophils with antioxidant compounds. Interestingly, a recent study performed on a mouse model of idiopathic ASD, called BTBR [67], showed that DEHP treatment in juvenile mice induced persistent inflammatory changes in both the brain and peripheral tissues, as well as behavioral dysregulation in adulthood, which were associated with decreased global DNA methylation levels [68]. These data suggest that environmental factors could contribute to ASD etiology by altering DNA methylation patterns, which, in turn, could alter the DNA binding of transcription factors related to the immune and inflammatory pathways.

Only one investigation included in the current systematic review investigated the epigenetic clock in peripheral blood, observing that ASD children had a biological age greater than their chronological age [46]. The epigenetic clock is a relatively new method for analyzing genome-wide DNA methylation data, allowing us to evaluate an individual’s biological age. When the difference between an individual’s biological and chronological age is positive, it is assumed that the individual has accelerated biological aging, and in turn an accelerated “epigenetic clock” [69]. In recent years, an accelerated epigenetic clock has been associated with several diseases, including cardiovascular diseases, diabetes, various types of cancer, and all-cause mortality, as well as psychiatric disorders both in children and in adults [70,71]. However, the study conducted by Neri de Souza Reis and co-workers is the first to link accelerated biological aging to ASD, and further studies would be needed to confirm whether the accelerated epigenetic clock is a hallmark feature of ASD children.

**Figure 3:**
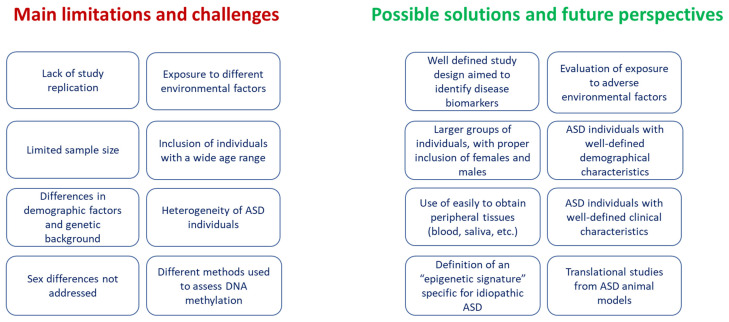
Limitations and challenges in the current literature on DNA methylation in ASD and possible solutions.

Several limitations need to be addressed in order to carry out a more exhaustive systematic review aimed at identifying the DNA methylation biomarkers of the clinical implications for the management of ASD children (Figure 3). Indeed, the current literature does not yet allow for the identification of valid DNA methylation biomarkers that are useful for the clinical management of ASD children. First, it should be emphasized that there are numerous papers published so far in which the relationship between DNA methylation and ASD has been investigated, but these studies were excluded from the current research because they did not meet the inclusion criteria established for this systematic review. One of the major criteria that was not met was the biological specimen in which the DNA methylation analysis was conducted, which, in most cases, was the brain. Another criterion that led us to exclude numerous articles was the age of the ASD patients, given that, in the majority of them, individuals with highly variable ages were recruited. Regarding the articles included in the systematic review, the main limitation preventing us from reaching a clear conclusion is that the most interesting results are related to studies that have not been replicated by independent research groups. Therefore, the data obtained until now need to be replicated in further studies. Several factors may have contributed to the lack of replication of the findings, including the different study designs, the often limited sample size of the ASD children included, demographic factors, the genetic background, exposure to different environmental factors, and the different methodologies used to evaluate DNA methylation levels. Another factor to be considered is that ASD is a highly heterogeneous disorder, and the inclusion of ASD individuals with different clinical presentations in the same study could introduce an important bias that does not allow us to detect specific ASD biomarkers. Moreover, further studies should be properly designed in order to evaluate sex differences. Indeed, it is becoming increasingly clear that males and females with ASD may have distinct epigenetic markers, and this issue has been considered in very few studies. Despite these limitations, the results of the current systematic review suggest that peripheral DNA methylation could provide biomarkers for ASD, even in young subjects. The usefulness of peripheral DNA methylation in the clinical management of several diseases, including various types of cancer, as well as neurological, immunological, and metabolic disorders, has been suggested in recent years, and some commercial kits for clinical use are already available [72]. Moreover, the recent development of the so-called “epigenetic signature” obtained through the evaluation of DNA methylation at the genome-wide level, which is implemented with computational algorithms, allowed for the diagnosis of 42 Mendelian neurodevelopmental disorders that are not always diagnosable with classical genetic tests [73,74]. Interestingly, some of these disorders are syndromes known to have autistic signs in their phenotypes, including Rett syndrome and Fragile X syndrome, and further studies could reveal their potential utility in the diagnosis and prognosis of idiopathic ASD. Similarly, it has recently been observed that the epigenetic signature of the postmortem brain tissues of syndromic ASD patients was similar to those observed in idiopathic ASD and was highly distinguishable from that of control subjects [75]. Further studies performed in peripheral tissues, including blood or saliva/buccal cells, will help us to understand whether the epigenetic signature of syndromic ASD could also be helpful for the detection and prognosis of idiopathic ASD, as well as for monitoring individual responses to pharmacological treatments. Additionally, the genes to be targeted in human subjects could be obtained by studies performed in ASD animal models. Indeed, several mouse models of ASD, in which the main ASD molecular and cellular phenotypes are present, including altered synaptic activity, neuronal migration abnormalities, and immune/inflammatory alterations, and which recapitulate the core symptoms of the disorder, are now available [12].

## 5. Conclusions

In conclusion, the current systematic review shows that the evaluation of methylation levels in DNA extracted from the peripheral tissues of ASD children could provide biomarkers with clinical utility in patient management. Indeed, some genes, including *OR2L13*, *BCL2*, *ZNF57*, and *SDHAP3*, as well as global DNA methylation levels, have been found to be altered in the peripheral tissues of ASD children when compared to age- and sex-matched TD individuals, potentially providing peripheral biomarkers for disease diagnosis. Moreover, the methylation of specific genes was found to be associated with phenotypical features of the disorder, e.g., the *RELN* and *ESR2* genes were associated with disease severity, the *NGF* gene was associated with mental retardation, and the *ST8SIA2* and *TGFB1* genes were associated with behavioral phenotypes. Furthermore, the data suggest that peripheral DNA methylation could provide sex-specific ASD biomarkers. However, the data obtained until now need to be replicated and confirmed in further studies in order to identify the DNA methylation biomarkers with clinical utility for ASD.

## Figures and Tables

**Table 1:** Main characteristics of the studies included in the systematic review.

Reference	Tissue Information	Sample Size	ASD Age (Range or Mean ± SD)	ASD Diagnosis and Severity	Methodology for DNA Methylation Analysis	Genomic Region Analysed	Main Findings
Alshamrani et al., 2023 [33]	Peripheral blood	28 ASD and 24 TD	7.5 ± 2.9	DSM-5, CARS	ELISA assay	Global methylation	Global DNA hypomethylation in ASD subjects.
Andrews et al., 2018 [34]	Peripheral blood	453 ASD and 515 TD	Between 3 and 5 years	ADOS, ADI-R	Illumina 450 K array	Genome-wide	No CpG sites reached EWAS threshold significance. Most DMPs were associated with the *CENPM*, *FENDRR*, *SNRNP200*, *PGLYRP4*, *EZH1*, *DIO3*, and *CCDC181* genes.
Aspra et al., 2022 [35]	Buccal cells	27 ASD and 15 TD	5.2 ± 1.9 years	ADI-R, SRS	Illumina 450 K array	Genome-wide	The hypermethylation of DMR is associated with the *ZFP57*, *CPXM2*, and *NRIP2* genes. The hypomethylation of DMRs is associated with the *RASGRF2*, *GSTT1*, *FAIM*, and *SOX7* genes.
Bahado-Singh et al., 2019 [36]	Neonatal dried blood spots	14 ASD and 10 TD	At birth (29 h–79 h after birth)	DSM-IV classification	Illumina 450 K array	Genome-wide	CpG methylation changes were found in 230 loci, associated with 249 genes, including some previously associated with ASD (*EIF4E*, *FYN*, *SHANK1*, and *VIM*). The best predictive CpG sites were associated with seven genes: *NAV2*, *OXCT1*, *LOC389033*, *MYL9*, *ALS2CR4*, *C19orf73*, and *ASCL2*.
Elagoz Yuksel et al., 2016 [37]	Peripheral blood	27 ASD and 39 TD	Between 22 and 94 months	DSM-IV-TR, CARS	MSRE-PCR	*OXTR* gene	Higher frequency of *OXTR* promoter hypomethylation in ASD.
Gallo et al., 2022 [38]	Peripheral blood	42 ASD	4.8 ± 2.0 years	DSM-5, ADOS-2	MS-HRM	*MECP2*, *OXTR*, *HTR1A*, *RELN*, *BCL-2* and *EN-2* genes	High maternal gestational weight gain associated with increased *BDNF* methylation. Lack of maternal folic acid supplementation and low *RELN* methylation associated with higher severity of ASD.
García-Ortiz et al., 2021 [39]	Peripheral blood	53 ASD and 45 TD	43.7 ± 11.2 months	DSM-5, ADI-R, ADOS-2	Pyrosequencing	LINE-1 regions, *NCAM1* and *NGF* genes	Decreased LINE-1 and increased *NCAM1* methylation in ASD; increased *NGF* methylation in ASD with mental regression during the first two years of life compared to TD and to ASD without mental regression.
Gui et al., 2020 [40]	Buccal swabs	51 infants, 10 of which developed ASD	Between 8 months and 2 years	MSEL, ADOS, ADI-R	Illumina 450 K array	Genome-wide	No global DNA methylation levels differences between children with and without ASD. The most associated CpG to ASD resided in *TUFT1*, *CYCS*, *SND1* and *CACNA2D1* genes.
Hannon et al., 2018 [41]	Neonatal dried blood spot	629 ASD and 634 TD	6.08 ± 3.24 days	WHO-ICD-10 diagnosis codes	Illumina 450 K array	Genome-wide	No CpG sites reach EWAS threshold significance. The most associated CpG to ASD resided in *RALY* gene. Significant association between increased polygenic burden for autism and methylomic variation at two specific loci in chromosome 8 close to *FAM167A* and *RP1L1* genes, respectively.
Hu et al., 2020 [42]	Peripheral blood	61 ASD and 66 TD	4.02 ± 2.83 years	DSM-5, CARS, ABC	qMSP	*HTR4* promoter gene	Decreased *HTR4* methylation in ASD. The difference was significant in males, but not in females. Higher methylation in females ASD compared to males ASD. No differences between females and males TD subjects.
Jasoliya et al., 2022 [43]	Peripheral blood	23 ASD, 23 FXSA, and 11 TD	Between 2 and 6 years	DSM-5, ADOS	EPIC array	Genome-wide	Two genes, *PAK2* and *FANCD2* differentially methylated between ASD and TD
Liang et al., 2019 [44]	Peripheral blood	5 pairs of ASD-discordant monozygotic twins; 5 pairs of ASD concordant ASD monozygotic twins; 30 pairs of sporadic patients with age- and sex-matched controls	Twins = 5.7 years Sporadic ASD = 4.46	DSM-5; ADOS	Illumina 450 K array, RRBS, pyrosequencing	Genome-wide, *SH2B1* gene.	Identified 2397 DMRs between discordant twins, including the *SH2B1* gene. Methylation of *SH2B1* increased in ASD-discordant monozygotic twins compared to concordant monozygotic twins and in sporadic ASD compared to control subjects.
Melnyk et al., 2012 [45]	Peripheral blood	68 ASD, 54 TD, 40 unaffected siblings	5.8 ± 2.1 years	DSM-IV, ADOS, CARS	HPLC	Global methylation	Decreased global methylation in ASD compared to TD and siblings.
Neri de Souza Reis et al., 2021 [46]	Peripheral blood	67 ASD	4.7 ± 1.3 years	DSM-IV; ICD-10; ADI-R, CARS; VABS	Illumina 450 K array	Epigenetic clock	Parental and intrauterine ASD risk factors moderated by age acceleration associated with Vineland total score. Moreover, authors calculate the epigenetic clock as a proxy of post-natal stress exposure, finding that children had a higher biological age than chronological age.
Stoccoro et al., 2022 [47]	Peripheral blood	58 ASD	4.35 ± 1.79 years	ADOS-2	MS-HRM	*MECP2*, *OXTR*, *HTR1A*, *RELN*, *BCL-2* and *EN-2* genes	Sex-related methylation differences: methylation levels of *MECP2*, *HTR1A*, and *OXTR* genes were connected to females, and those of *EN2*, *BCL2*, and *RELN* genes to males. Various maternal factors, including a lack of folic acid supplementation, were associated with high disease severity. *BDNF* methylation was associated with various ASD risk factors.
Wang et al., 2017 [48]	Peripheral blood	54 ASD and 54 TD	4.24 ± 0.98 years	DSM-IV, CARS, ABC	BSP	*ESR2* gene	Eight CpG sites were hypermethylated in ASD. Four CpG sites were positively associated with severe symptoms
Yang et al., 2022 [49]	Peripheral blood	30 ASD and 30 TD	Between 2 and 6 years	DSM-5, ADI-R	Pyrosequencing	*ST8SIA2* gene	Methylation levels of two CpG sites of the *ST8SIA2* gene were higher in ASD than in TD. One of these CpG sites positively correlated with the stereotyped behaviors of ASD children.
Zhao et al., 2018 [50]	Peripheral blood	42 ASD and 26 TD	4.07 ± 2.78 years	DSM-5, CARS, ABC	qMSP	*TGFB1*, *BAX*, *IGFBP3*, *PRKCB*, *PSEN2*, *CCL2*	Methylation levels of *TGFB1* were decreased in ASD compared to TD. *TGFB1* methylation was positively associated with the interaction ability score.

Abbreviations: ABC: Autism Behavior Checklist; ADOS-2: Autism Diagnostic Observation Schedule—Second Edition; ADI-R: Autism Diagnostic Interview—Revised; BSP: bisulphite sequencing; CARS: Childhood Autism Rating Scale; DMPs: differentially methylated regions; DSM: Diagnostic and Statistical Manual of Mental Illnesses; ELISA: enzyme-linked immunosorbent assay; FXSA: Fragile X syndrome; ICD-10: International Classification of Diseases, Tenth Revision; MS-HRM: Methylation-Sensitive High-Resolution Melting; MSEL: Mullen scales of early learning; MSRE-PCR, Methylation-sensitive restriction enzymes-PCR; qMSP, quantitative methylation-specific PCR; RRBS: reduced-representation bisulfite sequencing; VABS: Vineland Adaptative Behavior Scale.
